# Supplementary material for: Cooperative supramolecular polymers with anthracene‒endoperoxide photo-switching for fluorescent anti-counterfeiting
Source: Nat Commun. 2018 Sep 28;9:3977. doi: 10.1038/s41467-018-06392-x (PMC6162237; doi:10.1038/s41467-018-06392-x)
Supplement: Supplementary file 1 — Supplementary Information [file 41467_2018_6392_MOESM1_ESM.pdf]

Cooperative Supramolecular Polymers with Anthracene–Endoperoxide Photo-Switching for Fluorescent Anti-Counterfeiting

Z. Gao *et al.*

Supplementary Methods

Reagents and reactants: Copper iodide (CuI), 4-dimethylamino pyridine (DMAP), *N*-(3-(dimethylamino)propyl)-*N'*-ethylcarbodiimide hydrochloride (EDC•HCl), and benzyltriethylammonium chloride (TEBAC) were reagent grade and used as received. *Trans*-[Pt(PEt$_3$)$_2$I$_2$],[1] 4-ethynylaniline,[2] (*S*)-3,7-dimethyloctyl tosylate,[3] compounds **6**,[4] and **7** [5] were synthesized according to the previously literatures.

Theoretical calculations: all optimized structures were performed on G09 software packages (G09 A.02 [6] for $^1O_2$, and G09 B.01 [7] for **1** and **1**-O_2, respectively). All of the non-metallic elements (C, H, O, N, and P) were described by PBEPBE/6-31G computational method, whilst Pt atoms were described by Lanl2dz core potential. There are no imagery frequencies for the optimized geometries.

Mathematical models to characterize supramolecular polymerization process: for supramolecular polymerization of both **1** and **3**, deeper insights into the self-assembly processes are achieved by means of temperature-dependent CD spectra experiments. Non-sigmoidal curves are obtained by plotting the fraction of aggregated species (α_{agg}) against temperature for **1** and **3**, revealing the involvement of cooperative supramolecular polymerization process. To acquire the detailed thermodynamic parameters for the self-assembly processes, the normalized CD melting curves are fitted with the Meijer-Schenning-van der Schoot model.[8] In detail, the supramolecular polymerization can be divided into two separated steps: the nucleation and elongation regimes. In the elongation regime, the fraction of aggregated molecules φ_n is described by supplementary Equation 1:

$$\varphi_n = \varphi_{SAT}\{1 - exp[(-h_e) \times (T - T_e)/(R \times T_e^2)]\} \quad \textbf{(Supplementary Eq. 1)}$$

In this equation, h_e denotes the molecular enthalpy release due to the non-covalent supramolecular polymerization, T and T_e stand for the absolute temperature and elongation temperature, respectively. R represents the universal gas constant. φ_{SAT} is a parameter that is introduced to prevent the relation φ_n/φ_{SAT} surpassing the value of one.

In the nucleation regime, it means that at temperatures below the elongation temperature T_e. φ_n is described by supplementary Equation 2:

$$\varphi_n = K_a^{1/3} \times exp[(2/3K_a^{-1/3} - 1) \times h_e \times (T - T_e)/(R \times T_e^2)]$$

(**Supplementary Eq. 2**)

where K_a is the dimensionless equilibrium constant for the nucleation step at T_e.

Determination of the fluorescence resonance energy transfer (FRET) efficiency: the overlap integral (J) is calculated according to supplementary Equation 3.

$$J(\lambda) = \lambda^4 \times \mathrm{f_D}(\lambda) \times \varepsilon_\mathrm{A}(\lambda) \qquad \textbf{(Supplementary Eq. 3)}$$

where λ is the wavelength (cm), and $\varepsilon_\mathrm{A}(\lambda)$ is the molar extinction coefficient of FRET acceptor at wavelength λ. $\mathrm{f_D}(\lambda)$ is the fraction of fluorescence intensity of FRET donor.

The spectra overlap between the emission spectrum of donor **1** and the extinction spectrum of the acceptor **2** is shown in supplementary Figure 22. On this basis, J value is calculated to be 9.47×10^{11} M^{-1} cm^{-1} nm^4, which demonstrates the feasibility for FRET between **1** and **2** (see Supplementary Figure 23).

FRET efficiency (Φ_{ET}) is calculated according to supplementary Equation 4.[9]

$$\Phi_\mathrm{ET} = 1 - I_\mathrm{DA}/I_\mathrm{D} \qquad \textbf{(Supplementary Eq. 4)}$$

where I_{DA} and I_D are the emission intensity of FRET donor with and without the presence of FRET acceptor, respectively.

When **1** (40 μM) and **2** (4 μM) are mixed together in MCH, Φ_{ET} is calculated to be 80%.

Moreover, energy transfer rate constant (k_{ET}) is calculated according to supplementary Equation 5.[10]

$$\Phi_\mathrm{ET} = k_\mathrm{ET}/(k_\mathrm{ET} + \tau_\mathrm{D}^{-1}) \qquad \textbf{(Supplementary Eq. 5)}$$

where τ_D is the singlet state fluorescence lifetime of donor **1** in MCH.

When **1** (40 μM) and **2** (4 μM) are mixed together in MCH, k_{ET} is calculated to be 2.40×10^9 s^{-1}.

*Synthesis of compound **1***: compounds **7** (300 mg, 0.22 mmol), **8** (336 mg, 0.49 mmol),

CuI (~30 mg), and THF/Et_2NH (25 mL, 4 : 1, *v*/*v*) were stirred at room temperature for 12 hours. After removal of the solvent, the residue was extracted with H_2O/CH_2Cl_2 for three times. The combined organic extract was dried over anhydrous Na_2SO_4, and the solvent was removed with a rotary evaporator. The residue was purified by flash column chromatography (petroleum ether/CH_2Cl_2, 1 : 1, *v*/*v* as the eluent) to afford **1** as a yellow solid (494 mg, 90%). ^1H NMR (300 MHz, $CDCl_3$, room temperature, supplementary Figure 34) δ (ppm): 8.74 (dd, 4H), 7.63 (s, 2H), 7.50 (d, J = 8.7 Hz, 4H), 7.44 (dd, 4H), 7.32 (d, J = 8.7 Hz, 4H), 7.05 (s, 4H), 4.08–4.01 (m, 12H), 2.26–2.17 (m, 24H), 1.92–1.81 (m, 6H), 1.72–1.64 (m, 6H), 1.51–1.49 (m, 12H), 1.36–1.17 (m, 72H), 0.96–0.92 (t, 18H), 0.88–0.86 (d, 36H). ^{13}C NMR (75 MHz, $CDCl_3$, room temperature, supplementary Figure 35) δ (ppm): 207.22, 165.54, 153.35, 141.46, 135.23, 131.80, 131.68, 130.23, 128.35, 125.29, 124.81, 119.92, 105.83, 71.90, 67.85, 39.48, 39.39, 37.62, 37.46, 37.44, 36.48, 31.07, 29.95, 29.77, 28.12, 24.87, 24.85, 22.85, 22.75, 22.74, 19.74, 19.71, 19.69, 16.79, 16.61, 16.44, 8.58. ^{31}P NMR (121.5 MHz, $CDCl_3$, room temperature, supplementary Figure 36) δ (ppm): 11.55 (s, ^{195}Pt satellites, $^1J_{Pt-P}$ = 890.6 Hz). MALDI-TOF-MS *m*/*z*: $[M + H]^+$, 2464.4295 (supplementary Figure 37).

Synthesis of compound **3**: the synthetic procedure for **3** was similar to that for monomer **1**, except that compound **6** (200mg, 0.16 mmol) was used instead of **7**. The product was obtained as a yellow solid (355 g, 93 %). ^1H NMR (300 MHz, $CDCl_3$, room temperature, supplementary Figure 38) δ (ppm): 7.62 (s, 2H), 7.47 (d, J = 7.8 Hz, 4H), 7.30 (d, J = 7.8 Hz, 4H), 7.13 (s, 4H), 7.04 (s, 4H), 4.09–4.00 (m, 12H), 2.21–2.15 (m, 24H), 1.90–1.84 (m, 6H), 1.70–1.64 (m, 12H), 1.54–1.49 (m, 6H), 1.26–1.20 (m, 72H), 0.95–0.92 (t, 18H), 0.87–0.86 (d, 36H). ^{13}C NMR (75 MHz, $CDCl_3$, room temperature, supplementary Figure 39) δ (ppm): 165.52, 153.32, 141.41, 135.17, 131.58, 130.55, 130.18, 125.61, 125.29, 119.89, 105.79, 71.86, 67.80, 39.46, 39.37, 37.61, 37.45, 37.43, 36.46, 29.92, 29.82, 29.75, 28.10, 24.86, 24.83, 22.83, 22.74, 22.72, 19.72, 19.68, 16.60, 16.43, 16.25, 14.25, 8.48. ^{31}P NMR (121.5 MHz, $CDCl_3$, room temperature, supplementary Figure 40) δ (ppm): 10.88 (s, ^{195}Pt satellites, $^1J_{Pt-P}$ = 890.6 Hz). MALDI-TOF-MS *m*/*z*: $[M + H]^+$, 2364.3371 (supplementary Figure 41).

*Synthesis of compound **5***: dibromoanthracene (500 mg, 1.49 mmol), $Pd(PPh_3)_4$ (100 mg, 0.09 mmol) and CuI (50 mg, 0.26 mmol) were stirred in THF (15 mL) and $i$$Pr_2NH$ (15 mL). 1-Ethynyl-4-methoxybenzene (492 mg, 3.73 mmol) was added dropwise to the reaction mixture over 30 minutes. After stirring at 80 ºC for 12 hours, the reaction mixture was evaporated to remove the solvent, and the residue was extracted with H_2O/CH_2Cl_2 for three times. The combined organic extracts were dried over anhydrous Na_2SO_4, and the solvent was removed with a rotary evaporator. The residue was purified by flash column chromatography (petroleum ether/CH_2Cl_2, 3 : 1 *v*/*v* as the eluent) to afford compound **5** as an orange solid (523 mg, 80 %).[11] ^1H NMR (300 MHz, $CDCl_3$, room temperature, supplementary Figure 42) δ (ppm): 8.73–8.65 (m, 4H), 7.75–7.69 (m, 4H), 7.66–7.60 (m, 4H), 7.02–6.96 (m, 4H), 3.89 (s, 6H).

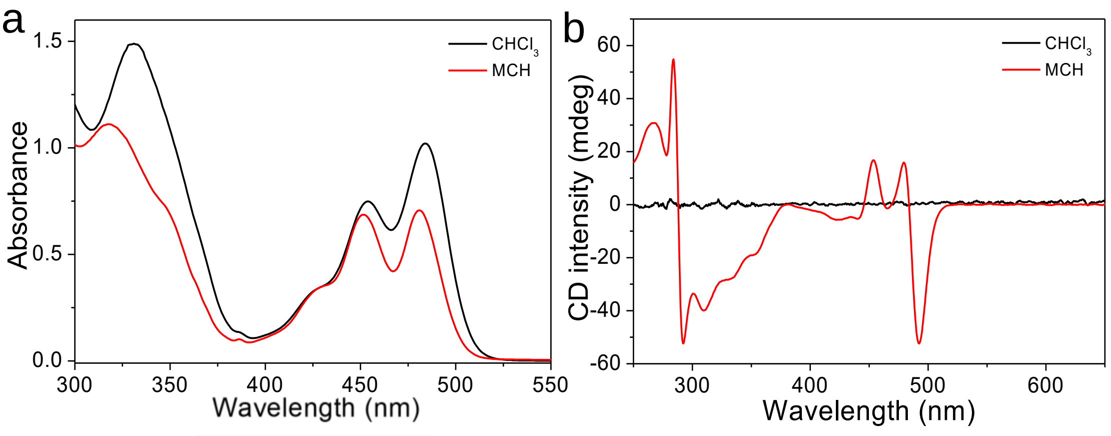

Supplementary Figure 1. a) UV–Vis and b) CD spectra of **1** (2.00×10^{-4} M, 298 K) in MCH (red lines) and $CHCl_3$ (black lines). At 298 K, no CD signals are detected for **1** in $CHCl_3$, because of the dominance of molecularly-dissolved state. In sharp contrast, strong Cotton effects are observed for the ^1L_a and ^1L_b bands in MCH.

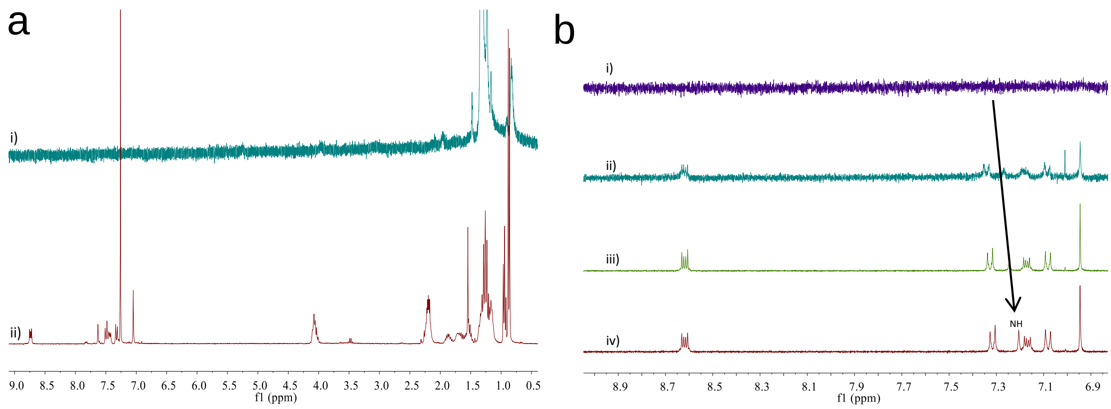

Supplementary Figure 2. a) ^{1}H NMR spectra (300 MHz, 298 K) of **1** in i) d_{12}-cyclohexane and ii) *d*-chloroform. b) Temperature-dependent ^{1}H NMR spectra of **1** in d_{12}-cyclohexane: i) 298 K, ii) 313 K, iii) 333 K, and iv) 343 K. The aromatic resonances of **1** in d_{12}-cyclohexane show broad signals at room temperature, which are in stark contrast to the well-resolved peaks observed in *d*-chloroform. Moreover, intermolecular hydrogen bonds exist between the neighboring amide units, as evidenced by the upfield shifting of amide resonanes upon elevating the temperature.

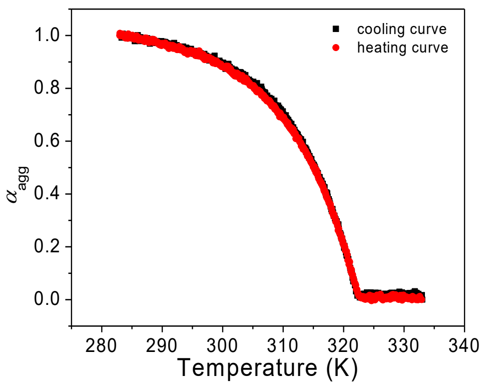

Supplementary Figure 3. Normalized CD intensity of **1** (2.00 × 10^{-4} M in MCH) at λ =486 nm observed in the cooling (black) and heating (red) processes at a rate of 40 K h^{-1}. The absence of heating–cooling hysteresis under the conditions illustrates the thermodynamic controlled supramolecular polymerization process.

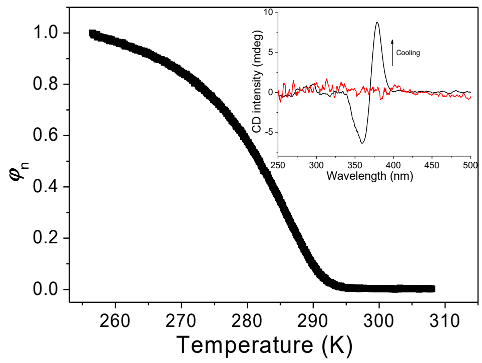

Supplementary Figure 4. Normalized CD signal at 378 nm as a function of temperature for monomer **3** (2.00 × 10^{-4} M in MCH). Inset: CD spectra of **3** at 300 K (red line) and 273 K (black line). It is evident that **3** adopts cooperative mechanism for supramolecular polymerization process.

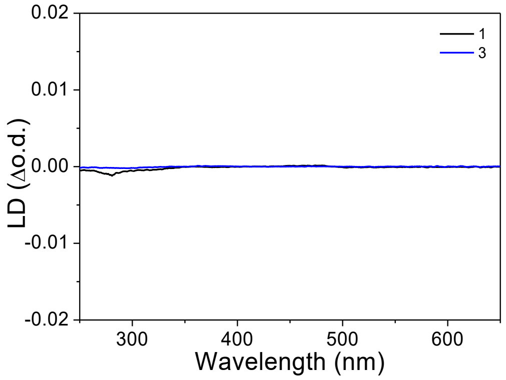

Supplementary Figure 5. LD signals of **1** and **3** (2.00 × 10^{-4} M in MCH, 283K). As can be seen, no LD signals are detected for the CD-active samples. Hence, the measured CD signals are real to reflect the supramolecular chirality, without the interference of CD artifacts.

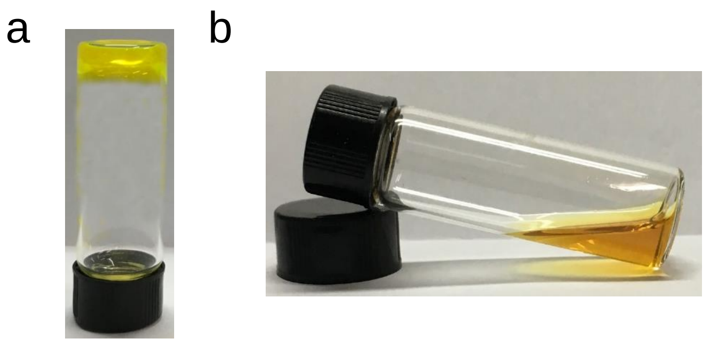

Supplementary Figure 6. Images of a) gel from monomer **1** (10 mM) and b) solution from monomer **3** (80 mM) in MCH at 293 K. For **1**, it shows the stronger gelation capability than that of **3** (critical gel concentration: ~8 mM for **1**, whilst no gel can be detected for **3** even increase concentration up to 80 mM).

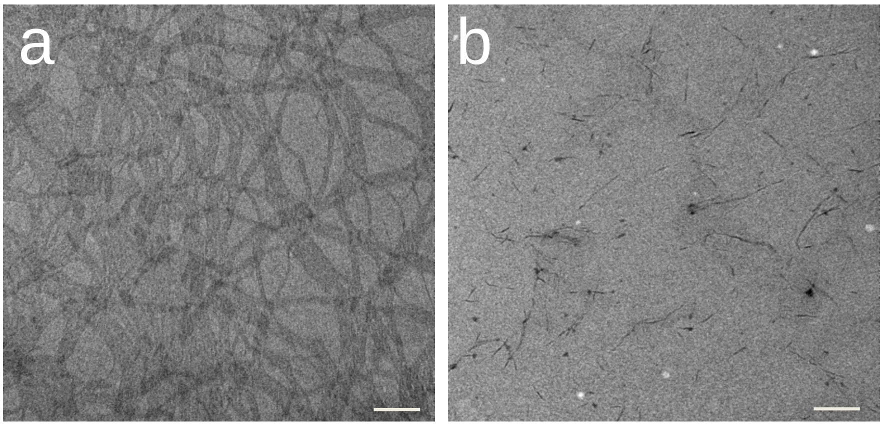

Supplementary Figure 7. TEM images of a) **1** and b) **3** on the copper grid (4.00×10^{-4} M). For **1**, long fibers are observed, which possess several microns in length and around 100 nm in width. Under the same conditions, **3** tends to form short bundles. Scale bars: 500 nm.

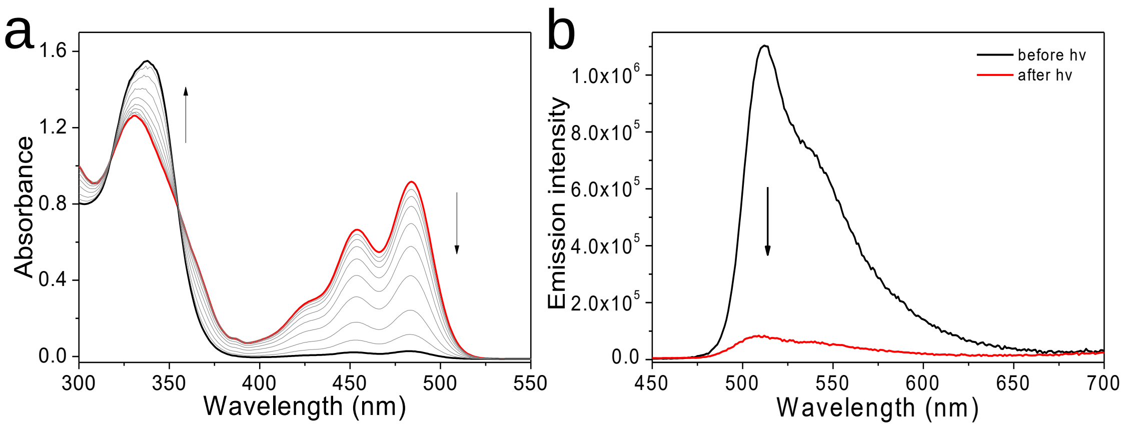

Supplementary Figure 8. a) UV–Vis and b) fluorescence spectra variations of **1** (2.00×10^{-4} M, $CHCl_3$) upon irradiation with 460 nm LED light. Upon photo-irradiation, both ^1L_a absorption (400–525 nm) and emission (475–650 nm) bands decrease significantly in their intensities.

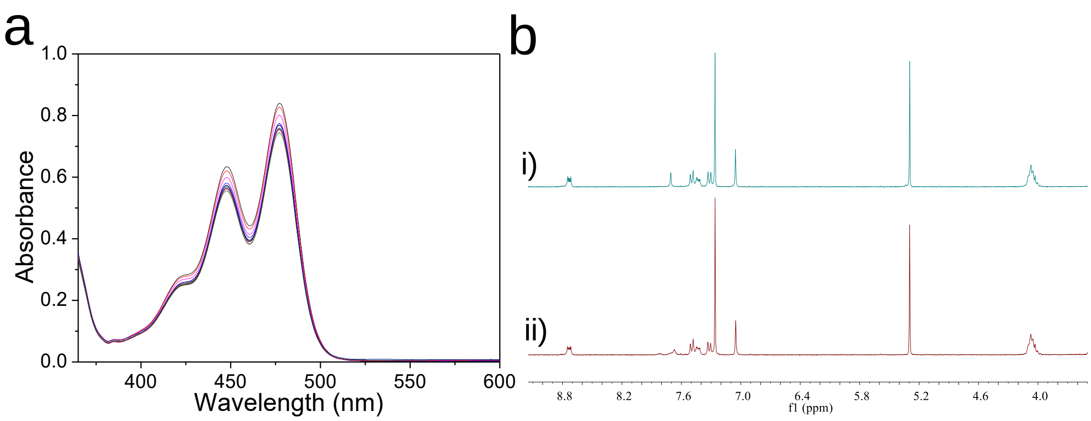

Supplementary Figure 9. a) UV–Vis spectra variation of **1** (2.00×10^{-4} M in $CHCl_3$) upon 460 nm LED light irradiation with the continuous bubbling of N_2. b) Partial ^1H NMR spectrum (300 MHz, 298 K) of **1** in N_2 atmosphere: i) before irradiation, ii) after light irradiation for 300 s.

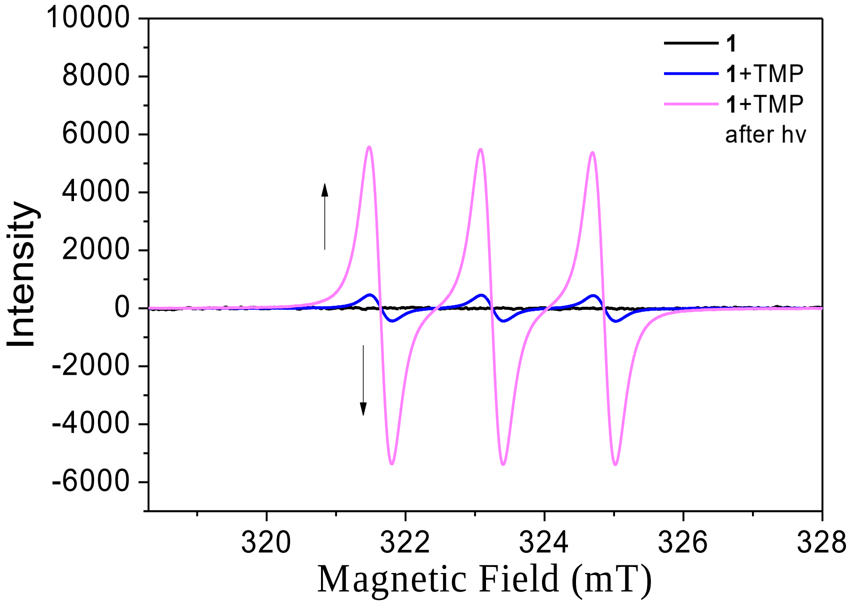

Supplementary Figure 10. EPR spectra of **1** (2.00×10^{-4} M in $CHCl_3$, black line), **1** with the presence of a trace amount of TMP before irradiation (blue line) and after 460 nm LED light irradiation for 300 s (pink line). When TMP serves as the singlet oxygen ($^1O_2$) capture, the EPR spectral pattern display three lines with equal intensity, which is characteristic for the formation of the paramagnetic nitroxide radical, TEMPO. It is rationalized that, $^1O_2$ generates *via* energy transfer between the triplet excited state of **1** and the surrounding O_2.

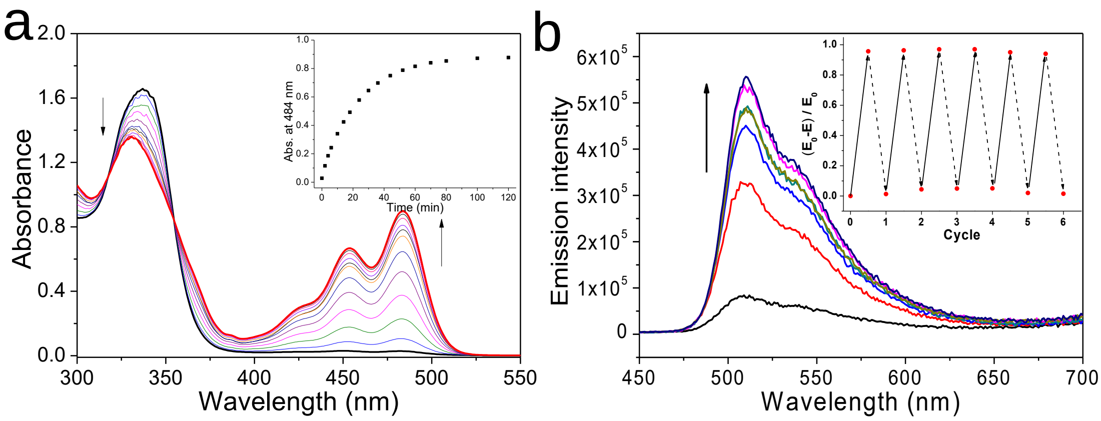

Supplementary Figure 11. UV–Vis and fluorescence spectra of **1**-O_2 (2.00×10^{-4} M in $CHCl_3$, λ_{ex} = 380 nm) upon standing at 293 K. Inset of a): UV–Vis spectra intensity of **1**-O_2 at 484 nm *versus* time. Inset of b): fluorescence spectra intensity at 517 nm upon multi-cycle irradiation and standing at 293 K. The anthracene signals are completely recoved in both absorption and emission spectra, indicates that **1**-O_2 can spontaneously convert back to **1** without any heat or light treatment. With the successive light irradiation and standing at room temperature, the reversible switching between **1** and **1**-O_2 can be repeatable for multiple cycles.

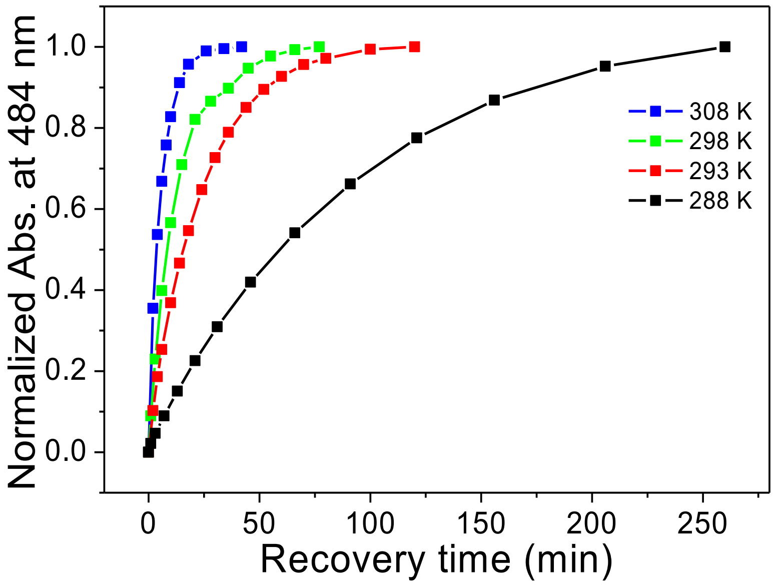

Supplementary Figure 12. Normalized UV–Vis spectra intensity of **1** (2.00×10^{-4} M) in $CHCl_3$ at 484 nm *versus* time at four different temperatures.

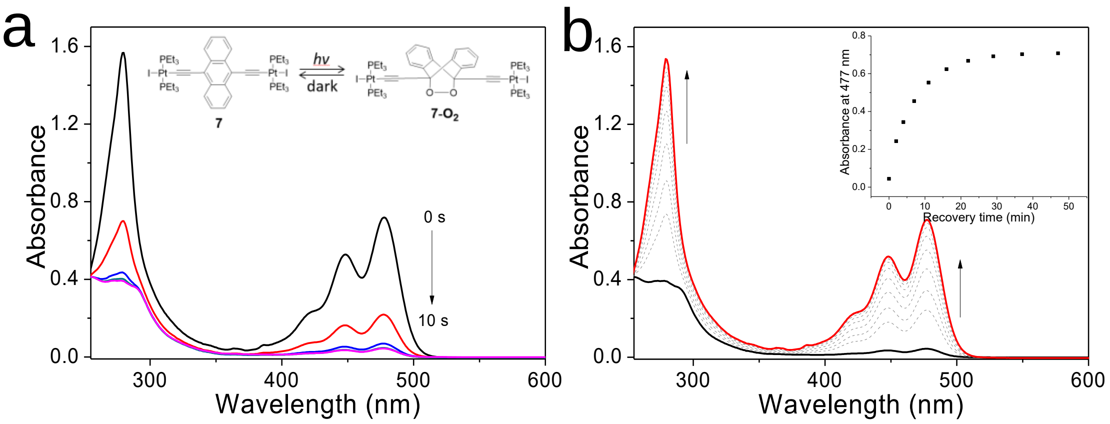

Supplementary Figure 13. UV–Vis spectra variation of **7** (2.00×10^{-4} M in $CHCl_3$) upon a) irradiating with 460 nm light, and b) restoring at room temperature at room temperature. The photo-switching behaviors of **7** are similar to those of **1**, suggesting that the peripheral non-conjugated units on **1** are unnecessary for photo-switching processes.

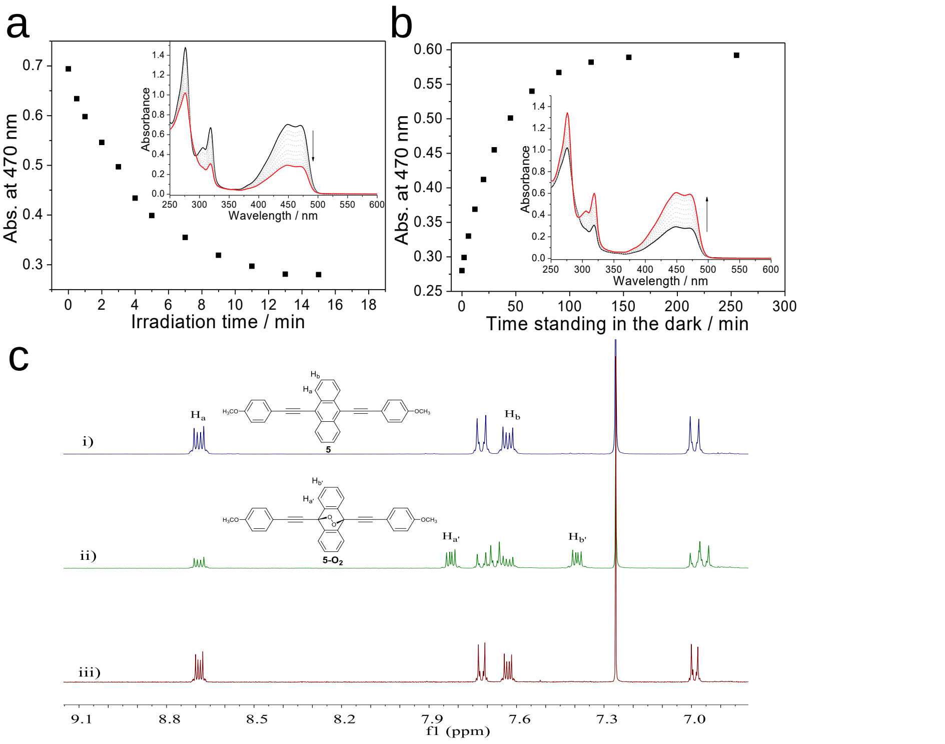

Supplementary Figure 14. UV–Vis spectra of **5** (2.00×10^{-4} M in $CHCl_3$) at 470 nm upon a) irradiating with 460 nm LED light, and b) standing at 298 K. c) Partial ^{1}H NMR spectra (2.00×10^{-3} M $CDCl_3$, 298 K) of **5**: i) before irradiation, ii) after irradiation for 60 min, and iii) heating at 50 ºC for 15 min. For photo-oxygenation of **5**, there is no complete conversion (~60% conversion), as evidenced by both UV–Vis and ^{1}H NMR measurements. Additionally, much longer irradiation time is required for **5** than that of **1** (800 s for **5** *versus* 20 s for **1** at 2×10^{-4} M, 298 K). For the deoxygenation process, the $t_{1/2}$ value for **5**-O_2 is determined to be 26 min, which is also slower than that of **1**-O_2 (8.6 min) under the same conditions.

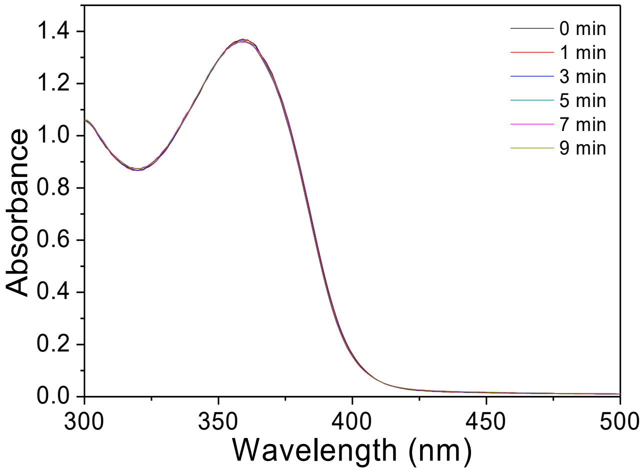

Supplementary Figure 15. UV–Vis spectra variation of **3** (2.00×10^{-4} M in $CHCl_3$) upon irradiation with 460 nm light. No conversion can be detected for **3** even with elongated irradiation time, suggesting the anthracene unit on **1** is crucial for the photo-switching process.

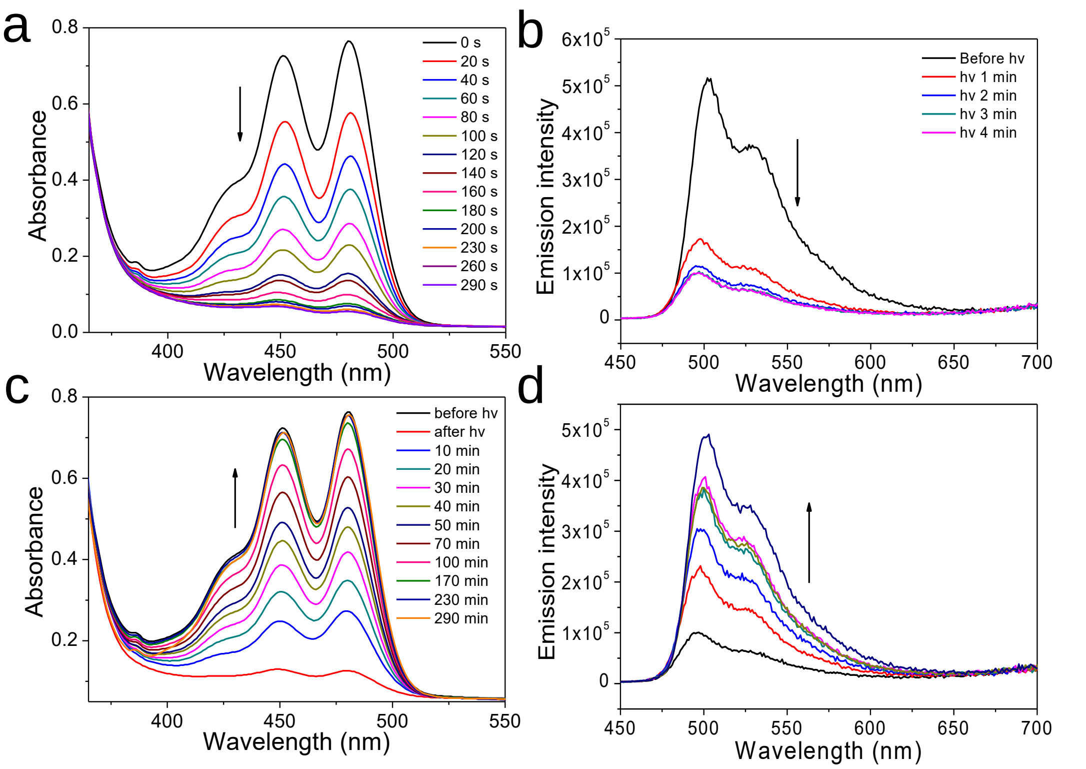

Supplementary Figure 16. UV–Vis and fluorescence spectra of **1** (2.00×10^{-4} M in MCH, λ_{ex} = 380 nm): a–b) upon irradiation with 460 nm light, c–d) upon standing at room temperature. Upon irradiation with 460 nm light, the absorbance and emission bands gradually decrease in their intensities. After standing at room temperature, the spectroscopic signals gradually recover. Hence, the reversible photo-switching of **1** also takes place in the supramolecular polymeric state.

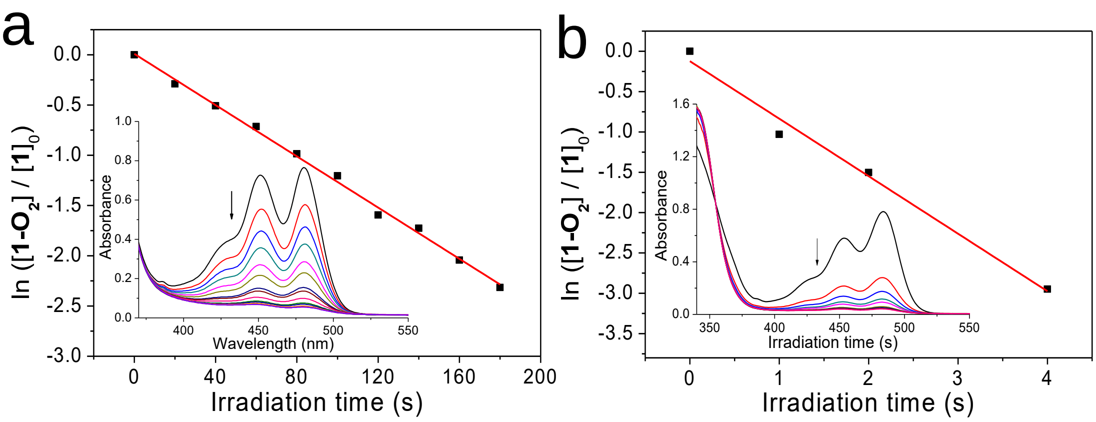

Supplementary Figure 17. Plots of ln([**1-O**$_2$]/[**1**]$_0$) *versus* irradiation time for the photo-oxygenation of **1** in a) MCH, and b) $CHCl_3$ (2.00×10^{-4} M, 293 K). The data were collected at the maximum value of each absorption. Inset: time-dependent UV–Vis spectra of **1** for 460 nm irradiation. The kinetics for the photo-oxygenation processes are different in the two solvents, as reflected by the slower observed rate constant (k_{obs}) in MCH than that of **1** in $CHCl_3$ solvent (k_{obs} = 0.0127 s^{-1} in MCH *versus* 0.71 s^{-1} in $CHCl_3$).

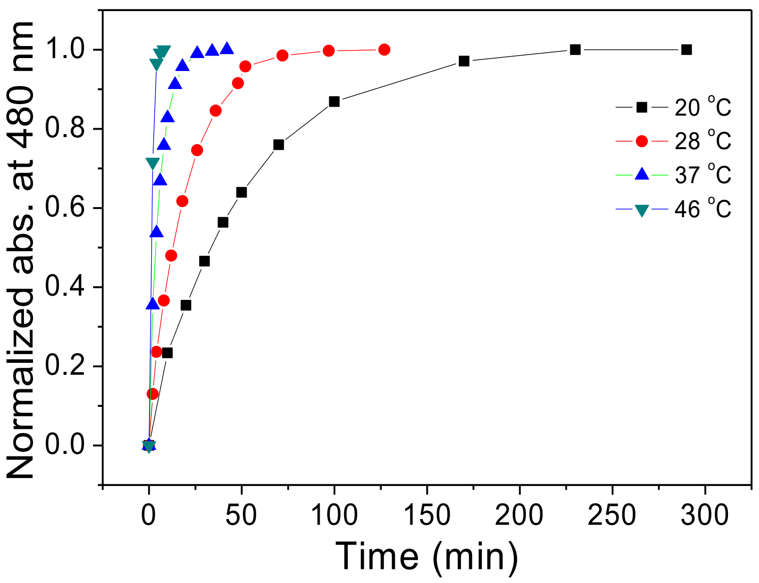

Supplementary Figure 18. Plots of the normalized absorbance intensity at 480 nm *versus* recovery time for the deoxygenation process from **1-O$_2$** to **1** in MCH. The half-life time ($t_{1/2}$) is determined to be 34 min at 293 K, which can be further modulated upon varying the temperature (from $t_{1/2}$ = 1.4 min at 319 K to 34 min at 293 K).

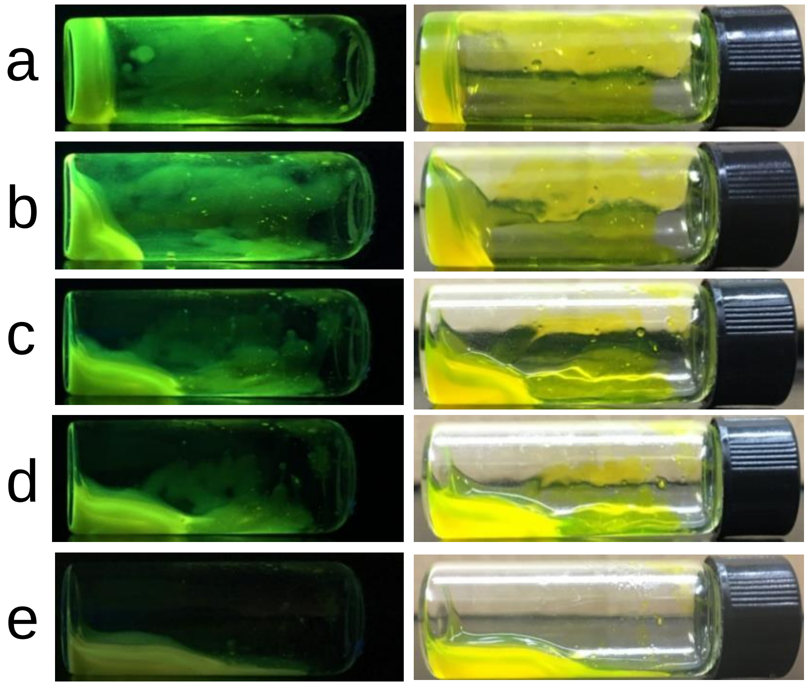

Supplementary Figure 19. Images of supramolecular gel from **1** (10 mM in MCH): a) before and after b) 30 s, c) 60 s, d) 120 s, e) 240 s irradiation with 460 nm LED light. Images were taken under 365 nm UV handhold lamp (left column) and natural light (right column). As can be seen, the gel began to collapse within 30 s, and completely converted to the "sol" state within 4 min. Notably, upon standing at room temperature, it returns back to the gel state.

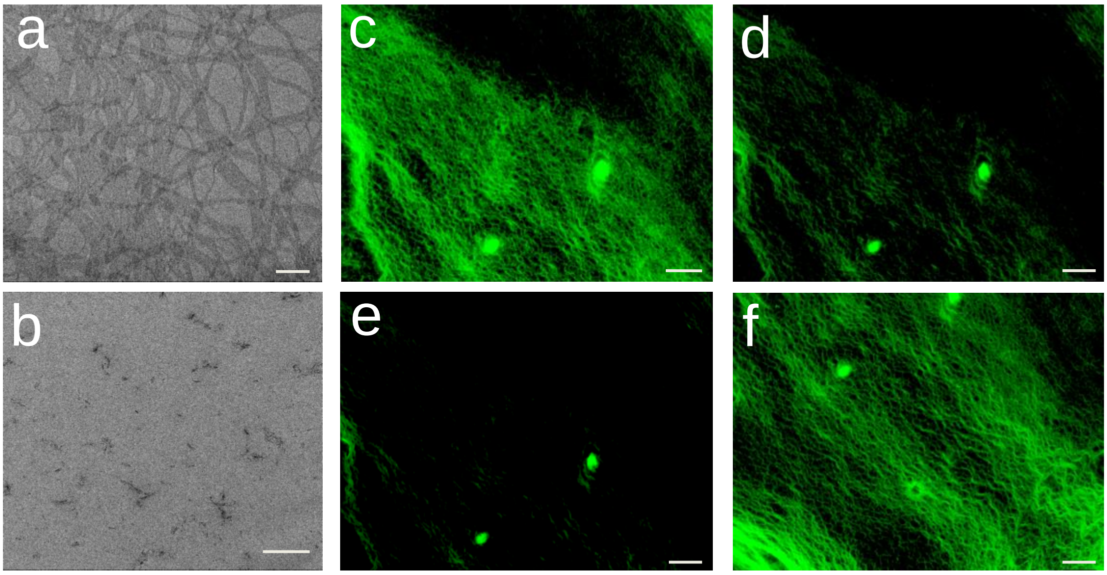

Supplementary Figure 20. TEM images of **1**: a) before irradiation (scale bar: 500 nm), and b) after irradiation with 460 nm light (scale bar: 1 μm). c–f) Fluorescence microscopy images of **1**: c) before irradiation, after d) 5 s, e) 15 s irradiation, and f) retore after room-temperature standing. Scale bars: 20 μm.

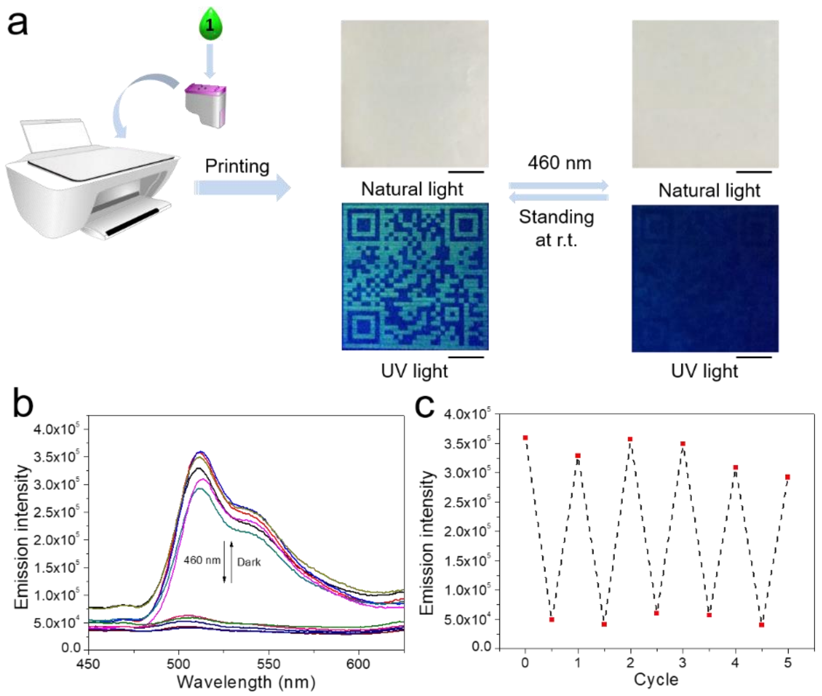

Supplementary Figure 21. a) The QR code on a paper (size: 5 × 5 cm) upon irradiating and standing at room temperature. It is obtained by employing **1** as the fluorescent anticounterfeiting ink. Scale bars: 1 cm. b) Fluorescence spectra (λ_{ex} = 380 nm) and c) intensity changes at 512 nm for the QR pattern as a function of irradiation/standing cycles. Thanks to the fully photo-switching reversibly of **1**, the QR information can be read and erased for multiple times.

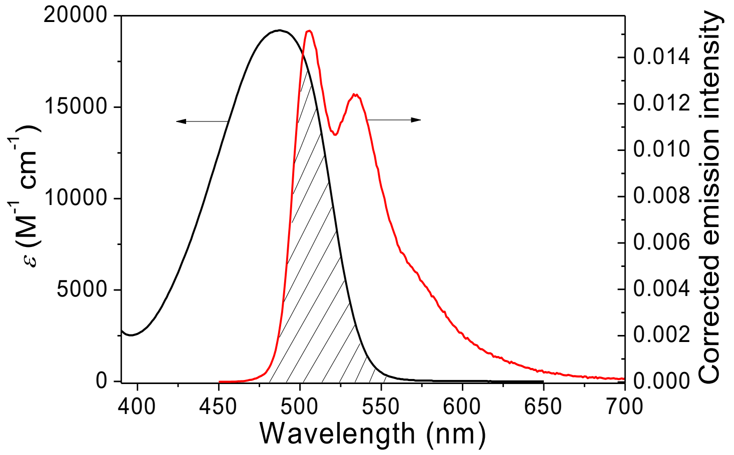

Supplementary Figure 22. The corrected emission spectrum $f_D(\lambda)$ of donor **1** and extinction coefficient spectrum $\varepsilon_A(\lambda)$ of acceptor **2**.

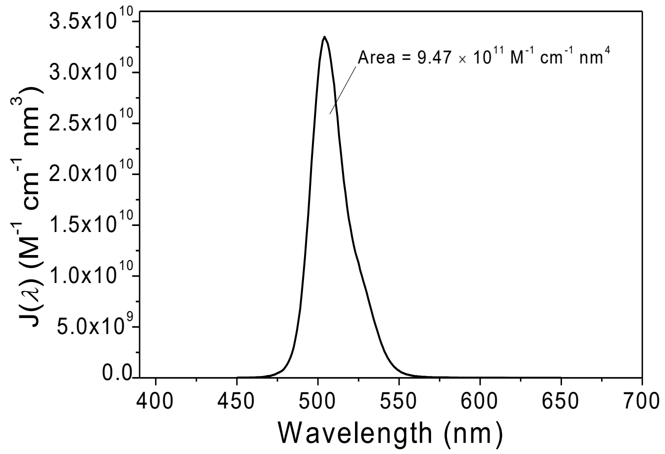

Supplementary Figure 23. Spectral overlap between the emission spectra of **1** and absorption spectra of **2**.

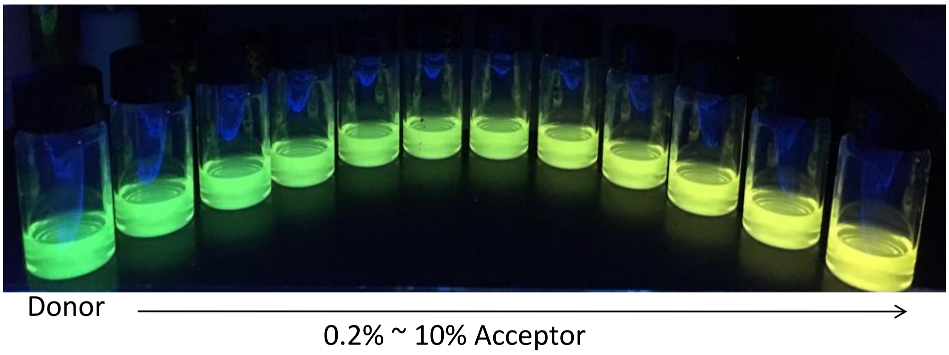

Supplementary Figure 24. Fluorescence images of FRET donor **1** with FRET acceptor **2** (0–10 mol%), which were taken under a 365 nm UV handhold lamp.

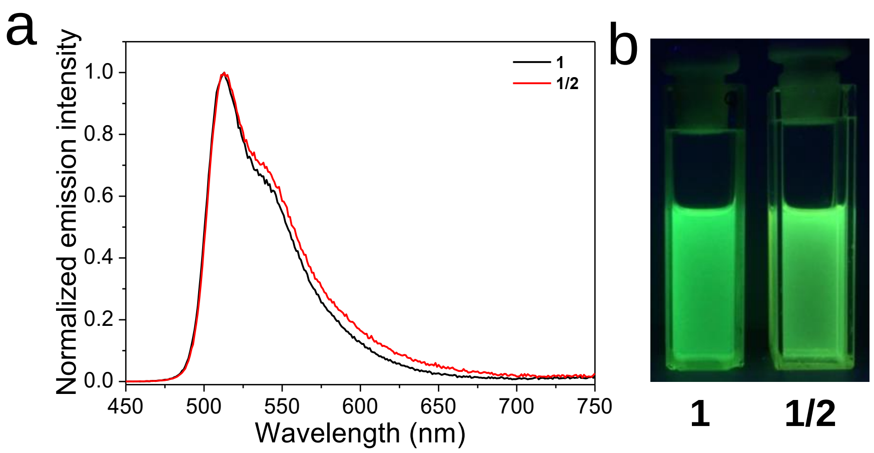

Supplementary Figure 25. a) Fluorescence spectra and b) images of **1** (4.00×10^{-5} M) and **1**/**2** (10 mol% of **2**) in $CHCl_3$. The emission spectrum of **1**/**2** in $CHCl_3$ is similar to that of **1**, suggesting the absence of FRET between **1** and **2** in $CHCl_3$.

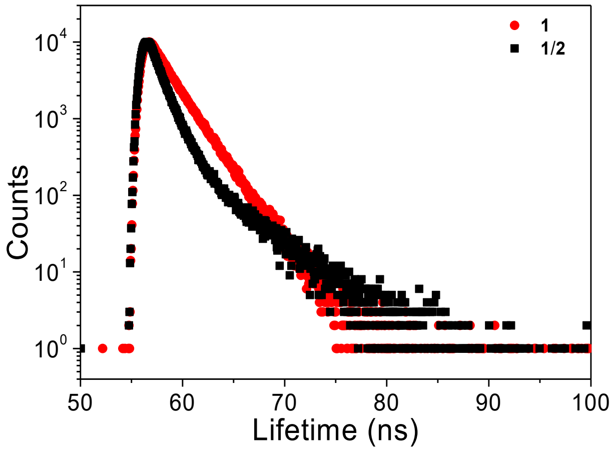

Supplementary Figure 26. Fluorescence decay profiles of donor **1** (red) and **1**/**2** (10 mol% of **2**, black). The fluorescence lifetime of **1** exhibits a single exponential with $\tau_D = 2.11$ ns. Moreover, the fluorescence lifetime for co-assembly **1**/**2** (10 mol% of **2**) exhibits a typically biexponential decay with $\tau_1 = 1.15$ ns and $\tau_2 = 4.11$ ns, respectively. The shortening of the donor lifetime from 2.11 ns to 1.15 ns originates from the FRET process from **1** to **2**.

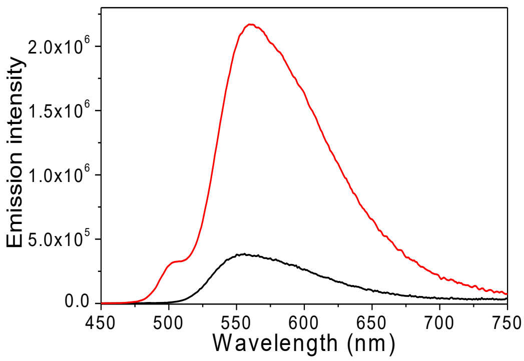

Supplementary Figure 27. Fluorescence spectra of **2** (4 μM, black line) and **1**/**2** (4 µM for **2** and 40 μM for **1**, red line). $\lambda_{ex} = 425$ nm.

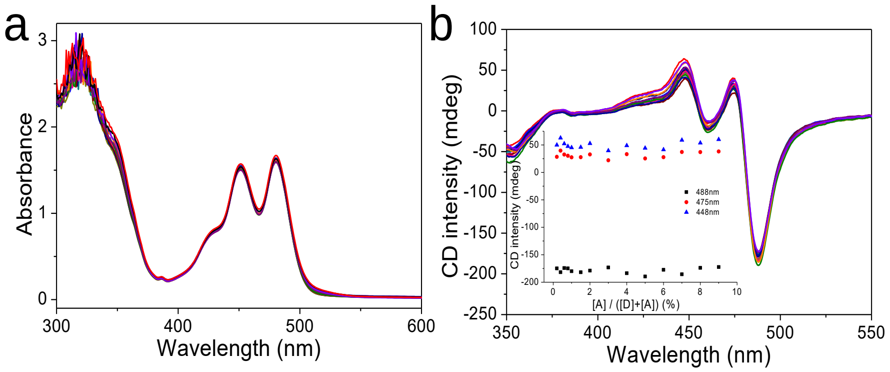

Supplementary Figure 28. a) UV–Vis and b) CD spectra of **1** (4.00×10^{-5} M), with 0–10 mol% of **2** in MCH. The absorbance bands of **1** at 395~500 nm was nearly constant upon gradually addition of **2**, indicating the absence of ground-state interactions between **1** and **2**. Moreover, the retained CD signals for **1**/**2** suggest that the encapsulation of **2** exerts minor impacts on the supramolecular ordering behaviors of **1**.

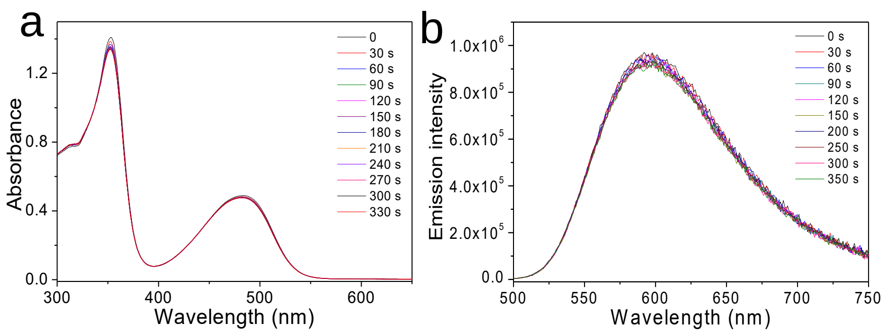

Supplementary Figure 29. a) UV–Vis absorption and b) fluorescence spectra of monomer **2** (2.00×10^{-5} M) in $CHCl_3$ upon irradiation with 460 nm light. No chemical conversion can be detected for **2** upon 460 nm light irradiation.

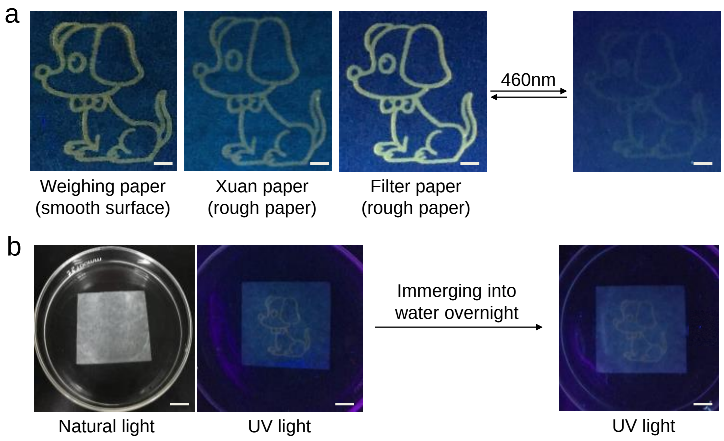

Supplementary Figure 30. a) Photographs of the photo-responsive fluorescent dog patterns derived from **1**/**2**, which are printed on various non-fluorescent papers (including the weighing paper, Xuan paper, and the filter paper). Scale bars: 5 mm. All of them display yellow emission colors, and show the excellent photo-responsive behaviors. b) Waterproof test for the printed pattern derived from **1**/**2**. Scale bars: 1 cm. It is apparent that the yellow fluorescence is still maintained, even after immerging the inkjet-printing pattern into water overnight.

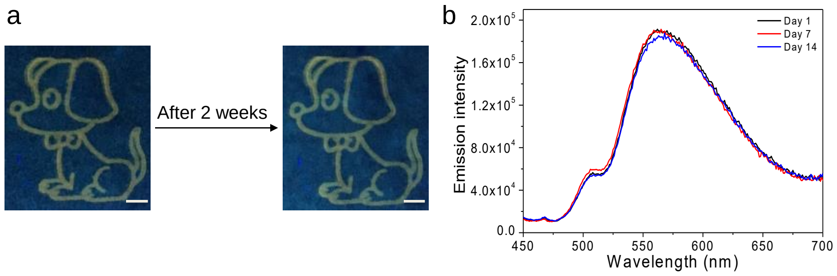

Supplementary Figure 31. a) Photographs and b) fluorescence spectra of the fluorescent dog patterns derived from **1**/**2** on day 1 and after 2 weeks. Scale bars: 5 mm. The printed dog pattern maintains yellow fluorescence after two weeks. On the basis of fluorescent measurements, it is also concluded that no obvious emission changes are found between the fresh-prepared and aged patterns.

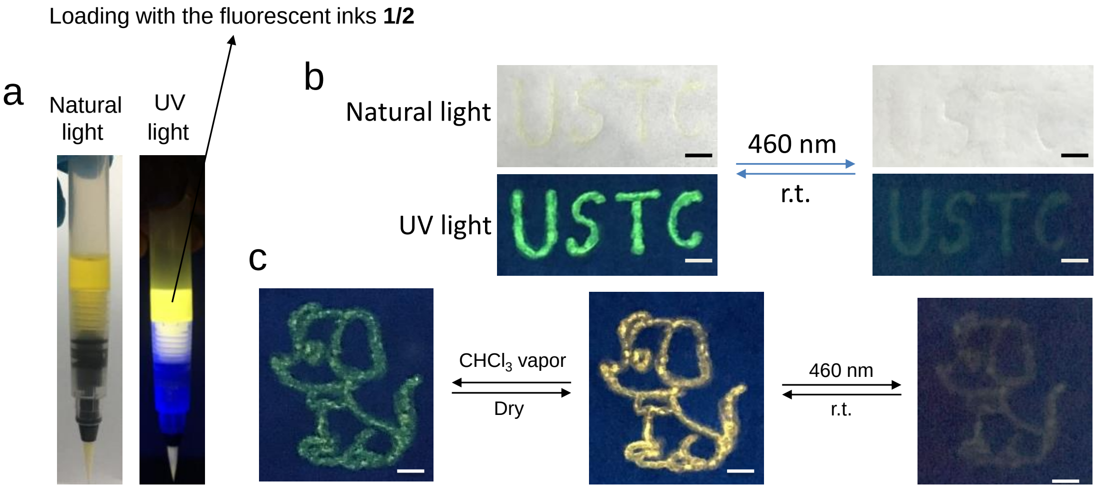

Supplementary Figure 32. Illustration of **1** and **1/2** as fluorescent anticounterfeiting inks on a paper writing letters and drawing patterns. a) Optical pictures of a fountain pen loaded with the fluorescent inks **1/2** (2.5 mol% of **2**). b) The MCH solution of **1** (1.00×10^{-4} M) as a fluorescent ink written with a word USTC on a piece of paper, which underwent 460 nm light irradiation and standing at room temperature (images taken under natural light and 365 nm handhold lamp). Scale bars: 1 cm. c) The MCH solution of **1/2** as a fluorescent ink drawing with a dog pattern on a paper and its responsiveness toward $CHCl_3$ vapor, as well as 460 nm light irradiation. Scale bars: 5 mm.

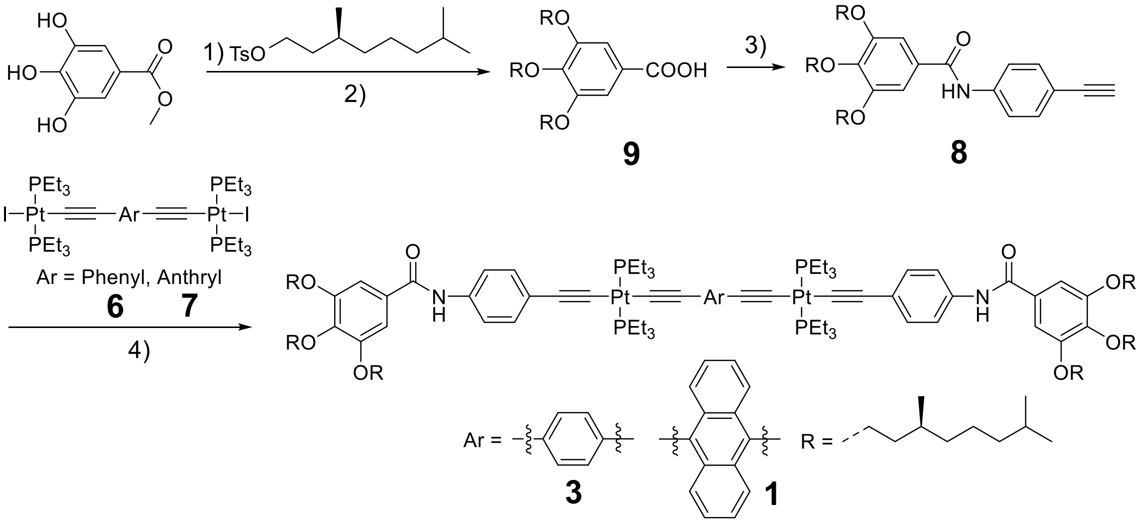

Supplementary Figure 33. Synthetic routes to the targeted monomers **1** and **3**. 1) CH_3CN, K_2CO_3, reflux; 2) ethanol, NaOH, reflux; 3) 4-ethynylaniline, EDC, DMAP, CH_2Cl_2; 4) CuI, Et_2NH/CH_2Cl_2, r.t..

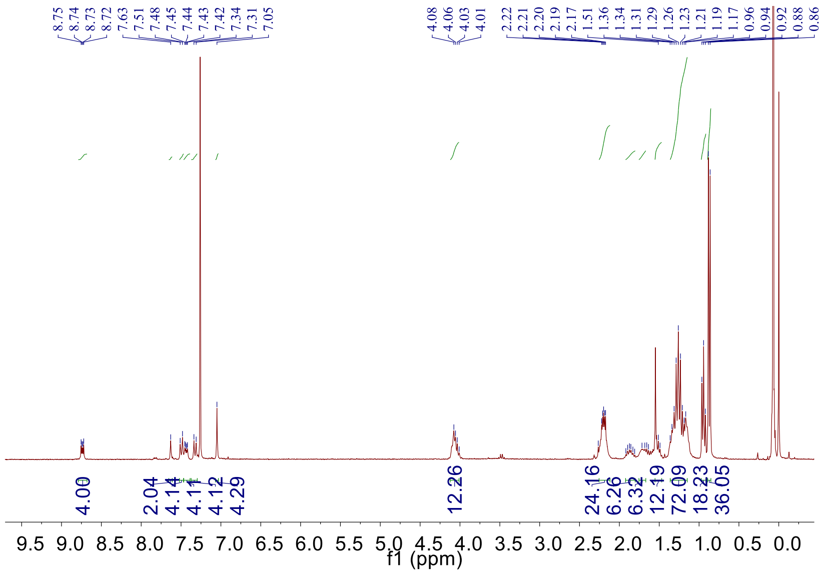

Supplementary Figure 34. ^{1}H NMR spectrum (300 MHz, $CDCl_3$, room temperature) of **1**.

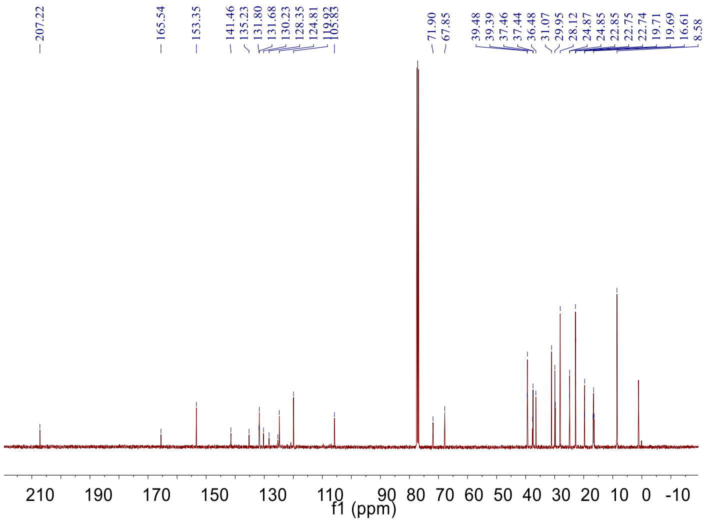

Supplementary Figure 35. ^{13}C NMR spectrum (75 MHz, $CDCl_3$, room temperature) of **1**.

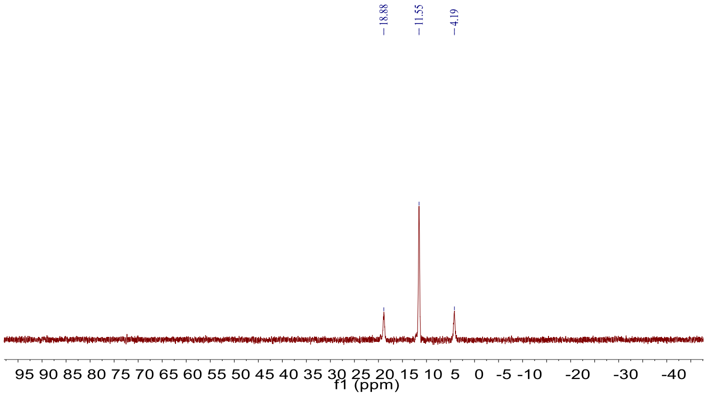

Supplementary Figure 36. ^{31}P NMR spectrum (121.5 MHz, $CDCl_3$, room temperature) of **1**.

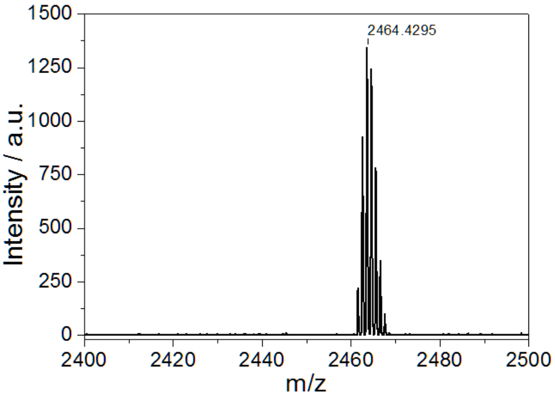

Supplementary Figure 37. MALDI-TOF mass spectrum of **1**.

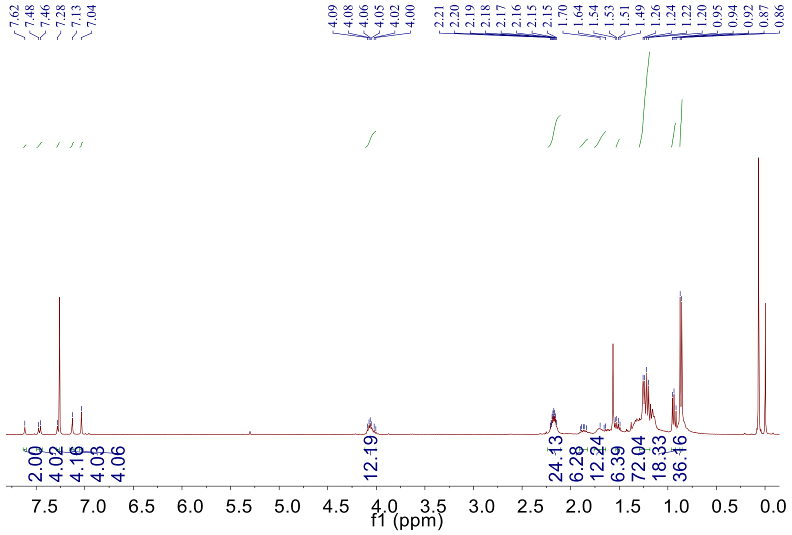

Supplementary Figure 38. ^{1}H NMR spectrum (300 MHz, $CDCl_3$, room temperature) of **3**.

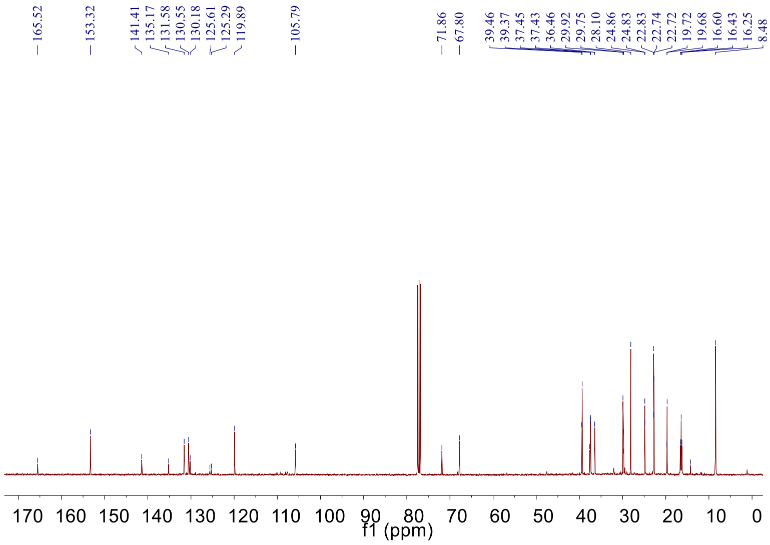

Supplementary Figure 39. ^{13}C NMR spectrum (75 MHz, $CDCl_3$, room temperature) of **3**.

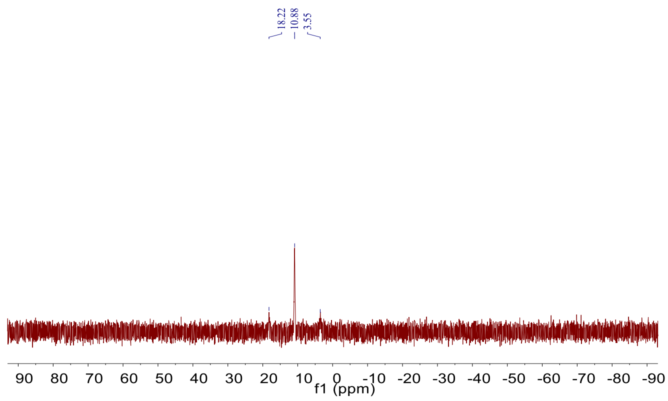

Supplementary Figure 40. ^{31}P NMR spectrum (121.5 MHz, $CDCl_3$, room temperature) of **3**.

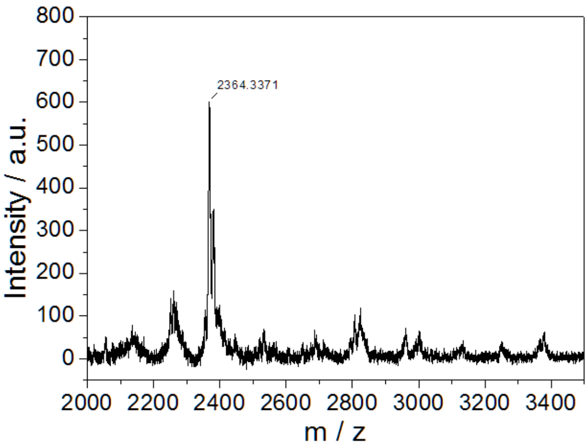

Supplementary Figure 41. MALDI-TOF mass spectrum of **3**.

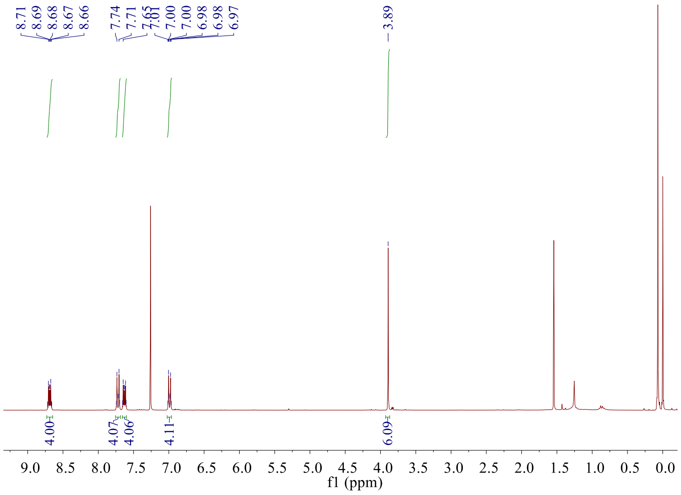

Supplementary Figure 42. ^{1}H NMR spectrum (300 MHz, $CDCl_3$, room temperature) of **5**.

Supplementary References:

1. Šembera, F. *et al*. Metal complexes with very large dipole moments: the anionic carborane nitriles 12-NC–$CB_{11}X_{11}$–(X = H, F, CH_3) as ligands on Pt(II) and Pd(II). *Inorg. Chem.* **55**, 3797–3806 (2016).
2. Maya, F., Chanteau, S. H., Cheng, L., Stewart, M. P. & Tour, J. M. Synthesis of fluorinated oligomers toward physical vapor deposition molecular electronics candidates. *Chem. Mater.* **17**, 1331–1345 (2005).
3. Helmich, F., Smulders, M. M. J., Lee, C. C., Schenning, A. P. H. J. & Meijer, E. W. Effect of stereogenic centers on the self-sorting, depolymerization, and atropisomerization kinetics of porphyrin-based aggregates. *J. Am. Chem. Soc.* **133**, 12238–12246 (2011).
4. Shi, E., Gao, Z., Yuan, M., Wang, X. & Wang, F. Self-assembly of benzothiadiazole-functionalized dinuclear platinum acetylide bolaamphiphiles for bio-imaging application. *Polym. Chem.* **6**, 5575–5579 (2015).
5. Khan, M. S. *et al*. Synthesis, characterisation and optical spectroscopy of platinum(II) di-ynes and poly-ynes incorporating condensed aromatic spacers in the backbone. *Dalton Trans.* 2377–2385 (2004).
6. Frisch, M. J. *et al. Gaussian 09, Revision A.02* (*Gaussian, Inc., Wallingford CT, 2009*).
7. Frisch, M. J. *et al. Gaussian 09, Revision B.01* (*Gaussian, Inc., Wallingford CT, 2010*).
8. Jonkheijm, P., van der Schoot, P., Schenning, A. P. H. J. & Meijer, E. W. Probing the solvent-assisted nucleation pathway in chemical self-assembly. *Science* **313**, 80–83 (2006).
9. Xu, Z. *et al*. Broad-spectrum tunable photoluminescent nanomaterials constructed from a modular light-harvesting platform based on macrocyclic amphiphiles. *Adv. Mater.* **28**, 7666–7671 (2016).
10. Liu, Y. *et al.* A srikaya-like light-harvesting antenna based on graphene quantum dots and porphyrin unimolecular micelles. *Chem. Commun.* **52**, 9394–9397 (2016).
11. Fudickar, W. & Linker, T. Why triple bonds protect acenes from oxidation and decomposition. *J. Am. Chem. Soc.* **134**, 15071–15082 (2012).
